# Churg-Strauss Syndrome following PTU Treatment

**DOI:** 10.1155/2009/504105

**Published:** 2009-03-08

**Authors:** R. A. M. Quax, A. J. G. Swaak, M. G. A. Baggen

**Affiliations:** ^1^Section of Endocrinology, Department of Internal Medicine, Erasmus Medical Centre Rotterdam, 3000 CA Rotterdam, The Netherlands; ^2^Department of Rheumatology, Ikazia Hospital, 3083 AN Rotterdam, The Netherlands; ^3^Department of Internal Medicine, Ikazia Hospital, 3083 AN Rotterdam, The Netherlands

## Abstract

Propylthiouracil (PTU) is a frequently prescribed drug in the treatment of hyperthyroidism. The use of PTU is, however, accompanied by numerous potentially serious side effects including vasculitis. PTU-related vasculitides can present as haematuria, pulmonary haemorrhage, or cutaneous lesion together with aspecific symptoms such as fever, myalgia, arthralgia, and fatigue. Cerebral involvement is seldom observed. We present a 49-year-old female with Graves' disease and asthma, who developed paresis of the proximal extremities, eosinophilia, pulmonary, and cutaneous lesions following treatment with PTU. A cerebral vasculitis consistent with Churg-Strauss syndrome (CSS) was suspected. Although cerebral involvement is seldom observed with PTU treatment, cerebral vasculitis should be considered in patients developing CNS symptoms.

## 1. Introduction

Numerous observations regarding the detection of a positive ANCA in patients treated with PTU in Graves' disease have been published [1–8]. The determination of antineutrophil cytoplasmic antibodies (ANCAs) is a highly specific and sensitive marker for the so-called ANCA-associated vasculitis. Vasculitis is a disease characterised by an inflammatory reaction in and around blood vessels. The vasculitides are classified on the basis of vessel size and the inflammatory reaction pattern. Using indirect immunofluorescence techniques (IFTs), two patterns of staining can be observed. Antiproteinase 3 (PR3) antibodies produce a diffuse cytoplasm pattern of staining in the IFT (c-ANCA). The presence of these antibodies is associated with Wegener's granulomatosis. A perinuclear pattern of immunofluorescence staining (p-ANCA) is a characteristic for the presence of anti-myeloperoxidase antibodies (MPO). These antibodies are linked with microscopic polyangiitis, but can also be found in Wegener's disease and Churg-Strauss syndrome. These diseases are taken together as ANCA-associated vasculitides. The exact mechanisms underlying these ANCA-associated vasculitides are unknown.

PTU-induced ANCA-associated vasculitides mainly relate to the renal, cutaneous, and pulmonary systems.

We describe a patient with Graves' hyperthyroidism, who developed a cerebral vasculitis consistent with Churg-Strauss syndrome after treatment with propylthiouracil (PTU).

## 2. Case Report

In July 2004, a 49-year-old woman was admitted to the Department of Internal Medicine with complaints of palpitations. Her past medical history was asthma, allergic diathesis, hay fever, and vitiligo. Laboratory examination demonstrated an elevated free T_4_ (30 pmol/L) and low TSH (<0.01 mIU/L). Iodine-123 scintigraphyshowed diffuse high uptake indicating Graves' disease. Treatment was started with thiamazole (30 mg per day). After two weeks of treatment with thiamazole, the patient developed a pruritis, and thiamazole was replaced by propylthiouracil (PTU) given in a dose of 100 mg three times a day. Levothyroxine was started at a dose of 87.5 *μ*g daily (block-and-replace therapy). Thereafter, she became euthyroid.

Five months after the start of PTU treatment, she developed a progressive loss of strength in her arms and legs. Her health deteriorated further with complaints of a painful abdomen, diarrhoea, and diffuse pruritis with skin rashes. Physical examination upon admission to the hospital showed petechiae, neurodermatitis, and nailfold lesions. A mild paresis of the proximal extremities was observed. Laboratory tests demonstrated an eosinophilia (4780 eosinophils/*μ*L and 44% eosinophils on differential white blood cell count). Liver enzymes and creatinine phosphokinase were normal. Serum creatinine was slightly elevated (111 *μ*mol/L) with an active urine sediment analysis showing traces of protein and microhematuria.

X-ray examination of the lungs revealed a diffuse interstitial pattern consistent with interstitial pneumonitis. In the following days, the patient developed fever and urticaria. Serological investigation revealed the presence of a positive ANCA with a titre of 1:2048. Anti-MPO (1:1280) and anti-PR3 (1:640) antibodies were also detected as measured by ELISA. Screening assays for antinuclear antibodies (ANAs/ENA/dsDNA) and rheumatoid factors (anti-CCP) were negative. Other causes for a vasculitis were excluded, like the presence of cryoglobulins or a hepatitis (screening assays for Hepatitides B and C, CMV, EBV, and HIV were negative). A systemic vasculitis was considered most likely. The differential diagnosis was of idiopathic hypereosinophilic syndrome, eosinophilia-myalgia syndrome, or parasitic infection. Although PTU was stopped immediately, there was progression of the neurological symptoms. She became totally bedridden due to progressive loss of strength of the proximal extremities. She also developed signs consistent with peripheral sensory polyneuropathy. The symptoms were more pronounced on the left side. Furthermore, and so far unnoticed, she demonstrated a positive Babinski sign on both sides. Cerebral magnetic resonance imaging revealed lesions in the parietal regions compatible with recent infarctions. Other causes of her fever and diarrhoea were excluded.

Under suspicion of a generalised vasculitis with cerebral extension, the patient was treated with methylprednisolone (1000 mg/d) for three days, and cyclophosphamide (15 mg/kg iv.) was started at four weeks time intervals. After 3 days, the methylprednisolone was changed in prednisolone (1 mg/kg/d). Under this treatment regime, neurological symptoms diminished rapidly, and the clinical signs of vasculitis including the nailfold lesions, skin rash, urticaria, and pruritis disappeared. Three weeks after onset of treatment, the ANCA titre was decreased to 1:1024. At that moment, laboratory values were all normalised. The ANCA titre further decreased to 1:64 after three months and became negative after 1 year until now.

We classified our patient as Churg-Strauss syndrome with cerebral involvement induced by the use of propylthiouracil (see Table 1).

Two years after initial presentation, she is in clinical good condition using 7.5 mg prednisone and 150 mg azathioprine daily.

## 3. Discussion

The development of ANCA following treatment with antithyroid medication has been described numerous times. In a large cross-sectional study [2] (*n* = 407), a high prevalence of a positive ANCA was described in patients with Graves' disease using antithyroid drugs as compared with euthyroid controls. In patients with Hashimoto's disease, there was no significant higher prevalence of ANCA. Moreover, the development of a positive ANCA was associated with propylthiouracil usage to a greater extent than with carbimazole, highlighting that the autoimmune disease itself is not (fully) responsible for the development of ANCA. In a small cross-sectional study [3] (*n* = 30), 27% of patients on long-term antithyroid medication were ANCA positive. These results were also confirmed in another retrospective study [4]; anti-MPO antibodies were detected in 25% of the patients treated with PTU, but only 3% of the patients treated with thiamazole. Data about the prevalence of a positive ANCA in patients with hyperthyroidism before the start of the treatment are scarce. In two of these studies [5, 6], the close relationship between the treatment with PTU and development of ANCA could be established. No correlation was detected between treatment with methimazole and ANCA positivity. A positive ANCA could only be demonstrated in the PTU treated patients. In children with Graves' disease, anti-MPO antibodies were found in 7% of patients before treatment. Of those patients treated with PTU, 64% developed anti-MPO antibodies (mean duration of PTU treatment 4.0 ± 3.6 yr) while none of the patients treated with thiamazole developed autoantibodies [7]. These cited observations strongly suggest that antithyroid treatment, specifically PTU, is associated with the development of ANCA.

However, an important question to solve is whether every patient who develops these kinds of antibodies will also develop an ANCA associated vasculitis.

Most of the literature concerning PTU-induced ANCA-associated vasculitides has been published as case reports. After the first publication by Dolman et al. [8] in 1993, numerous case reports of PTU-induced vasculitis have been described, with a wide spectrum of clinical presentation (see Table 2).

Renal involvement is mostly characterised by haematuria, proteinuria, or oliguria [9–25]. Renal biopsies showed two patterns, either pauci-immune necrotizing glomerulonephritis or pauci-immune crescentic glomerulonephritis.

Skin involvement as a cutaneous ANCA-associated vasculitis has been reported several times, with clinical signs as purpura, ulcerating skin, and erythematous lesions [8, 11, 14, 15, 18, 22, 26–30]. Skin biopsy mostly revealed the picture of a leucocytoclastic vasculitis [27, 28].

Pulmonary involvement is highly prevalent, although only one case was proven histologically [10]. In two other reports, lung biopsy only confirmed the presence of hemosiderin filled macrophages [20, 31]. Nevertheless, as dyspnoea, haemoptysis, or respiratory failure clearly improved on withdrawal of PTU or were directly correlated with histological proven vasculitis elsewhere, these clinical features are strongly indicative of pulmonary vasculitis [9, 10, 13, 17, 20–23, 25, 26, 31]. Other frequently observed symptoms are arthralgia, fever, myalgia, and scleritis.

To our knowledge, no literature is known about the use of antithyroid drugs and development of CSS. Even more so, the presence of eosinophilia is never mentioned in previous reports.

Recently, the first patient with a cerebral vasculitis following PTU treatment was described [1]. In contrast with our case report, this patient was only MPO-ANCA positive. Our patient developed central nervous system symptoms after using PTU for six months (versus 3 years in the other patient). The neurological symptoms of our patient consisted of a progressive loss of muscle strength, positive Babinski signs, and a sensory polyneuropathy, whereas the neurological symptoms of the other patient consisted of cognitive deficits. Signs of recent cerebral infarction were present in our patient. In both patients, the outcome improved after discontinuation of the PTU and the start of immunosuppressive therapy.

Despite accumulating reports demonstrating ANCA-associated vasculitis following treatment with antithyroid drugs, most ANCA-positive patients do not have clinical manifestations of vasculitis [2–7]. This discrepancy suggests that other factors are of importance in the development of vasculitis besides ANCA positivity.

Patients developing PTU-induced ANCA-associated vasculitis have been shown to have a significant higher titre and affinity of anti-MPO antibodies as compared with ANCA-positive patients without developing vasculitis [21]. Gao and coworkers found significantly decreased affinity and/or titre of anti-MPO antibodies after cessation of PTU and initiation of immunosuppressive therapy in patients with PTU-induced ANCA-associated vasculitis [22, 32]. Therefore, the titre and affinity of anti-MPO antibodies might be clinical markers for the development of PTU-induced ANCA-associated vasculitis.Further research is needed to establish this association.

## 4. Conclusion

Antithyroid drugs, especially PTU, are associated with the development of ANCA. Not every patient developing ANCA will develop a vasculitis, given the discrepancy between the prevalence of PTU-induced ANCA and clinically evident vasculitis. Consequently, screening of patients using PTU is not recommended. Further research is needed to asses if titre and affinity of anti-MPO antibodies can be used to predict the risk of developing vasculitis following treatment with antithyroid drugs. Patients using antithyroid drugs in general and PTU in particular should be closely monitored for signs of vasculitis, including central nervous system symptoms.

## Figures and Tables

**Table 1:** Criteria for the classification of Churg-Strauss syndrome (CSS). The presence of four or more of these criteria yields a sensitivity of 85% and a specificity of 99.7% for CSS according to the American College of Rheumatology (ACR).

ACR criteria	Present in our patient
Asthma	Yes
Eosinophilia >10% on differential white blood cell count	Yes
Mononeuropathy or polyneuropathy	Yes
Migratory or transient pulmonary opacities detected radiographically	Yes
Paranasal sinus abnormalities	No
Biopsy containing a blood vessel showing the accumulation of eosinophils in extravascular areas	—

**Table 2:** Most frequent observed clinical symptoms in PTU-induced ANCA-associated vasculitis. In total, 61 well-described patients were studied.

Syndrome	Frequency	Percentage (%)
Renal involvement	44	72
Fever	26	43
Arthralgia	22	36
Pulmonary involvement	16	26
Cutaneous lesions	14	23
Myalgia	11	18
Eye involvement	7	11
Pericardial effusion	2	3
Central nervous system	1	2
